# Adipokine Imbalance in the Pericardial Cavity of Cardiac and Vascular Disease Patients

**DOI:** 10.1371/journal.pone.0154693

**Published:** 2016-05-03

**Authors:** Atlanta G. I. M. Elie, Pia S. Jensen, Katrine D. Nissen, Ilvy M. E. Geraets, Aimin Xu, Erfei Song, Maria L. Hansen, Akhmadjon Irmukhamedov, Lars M. Rasmussen, Yu Wang, Jo G. R. De Mey

**Affiliations:** 1 Dept. Cardiovascular and Renal Research, Institute of Molecular Medicine, University of Southern Denmark, Odense, Denmark; 2 Dept. Clinical Biochemistry and Pharmacology, Odense University Hospital, Odense, Denmark; 3 Cardiovascular Research Institute Maastricht, Maastricht, the Netherlands; 4 State Key Laboratory of Pharmaceutical Biotechnology and Dept. Pharmacology and Pharmacy, Li Ka Shing Faculty of Medicine, The University of Hong Kong, Hong Kong, China; 5 Dept. Cardio-Thoracic and Vascular Surgery, Odense University Hospital, Odense, Denmark; 6 Centre for Individualized Medicine in Arterial Diseases (CIMA), Odense University Hospital, Odense, Denmark; Indiana University School of Medicine, UNITED STATES

## Abstract

**Aim:**

Obesity and especially hypertrophy of epicardial adipose tissue accelerate coronary atherogenesis. We aimed at comparing levels of inflammatory and atherogenic hormones from adipose tissue in the pericardial fluid and circulation of cardiovascular disease patients.

**Methods and Results:**

Venous plasma (P) and pericardial fluid (PF) were obtained from elective cardiothoracic surgery patients (n = 37). Concentrations of leptin, adipocyte fatty acid-binding protein (A-FABP) and adiponectin (APN) were determined by enzyme-linked immunosorbent assays (ELISA). The median concentration of leptin in PF (4.3 (interquartile range: 2.8–9.1) μg/L) was comparable to that in P (5.9 (2.2–11) μg/L) and these were significantly correlated to most of the same patient characteristics. The concentration of A-FABP was markedly higher (73 (28–124) versus 8.4 (5.2–14) μg/L) and that of APN was markedly lower (2.8 (1.7–4.2) versus 13 (7.2–19) mg/L) in PF compared to P. APN in PF was unlike in P not significantly related to age, body mass index, plasma triglycerides or coronary artery disease. PF levels of APN, but not A-FABP, were related to the size of paracardial adipocytes. PF levels of APN and A-FABP were not related to the immunoreactivity of paracardial adipocytes for these proteins.

**Conclusion:**

In cardiac and vascular disease patients, PF is enriched in A-FABP and poor in APN. This adipokine microenvironment is more likely determined by the heart than by the circulation or paracardial adipose tissue.

## Introduction

Obesity promotes atherosclerosis [1, 2]. Hypertrophic chronically inflamed adipose tissues produce less signaling molecules with vascular protective actions such as adiponectin (APN) [3, 4] and more hormones with inflammatory and atherogenic effects such as leptin [5] and adipocyte fatty acid-binding protein (A-FABP) [6]. The circulating hormonal levels of these adipokines are likely dominated by the large subcutaneous and abdominal visceral adipose tissue depots [7].

Local accumulation of fat on the outer surface of the heart around the coronary arteries (epicardial adipose tissue (EAT) [8, 9]) is associated with coronary artery disease (CAD) in not only obese but also non-obese patients [10–15]. This indicates local paracrine effects of adipokines on coronary arteries [8, 16, 17]. Paired comparisons of EAT with subcutaneous or abdominal visceral adipose tissue biopsies revealed differences in fatty acid composition [18], expression and release of chemokines and inflammatory cytokines along with retention of inflammatory cells [15, 19] and in expression and secretion of adipokines [14, 15, 17, 20].

The composition of the pericardial fluid (PF) surrounding the heart has been proposed to be comparable to that of the interstitium of the heart [21, 22]. Several angiogenic growth factors, cytokines, natriuretic peptides and classical vasoactive hormones are present at higher concentration in the PF than in the circulating plasma (P) of patients with ischemic heart disease or heart failure [21, 23–25].

Here we tested the hypothesis that the pericardial cavity of cardiovascular disease (CVD) patients is a unique adipokine microenvironment. To this end we compared the levels of leptin, A-FABP and APN in the PF and P of cardiothoracic surgery patients.

## Material and Methods

### Subjects and sample collection

The study was performed in accordance with institutional guidelines and was approved by the Medical Ethical Committee of the Region of Southern Denmark (S-20100044). Investigations were performed conform the principles outlined in the Declaration of Helsinki and informed written consent was obtained from all patients. Between September 2013 and January 2014 consecutive patients who underwent elective coronary artery bypass grafting (CABG) or cardiac valve replacement surgeries were enrolled. Clinical information was obtained from the patients’ medical records. On the day before surgery, peripheral venous blood was drawn into pyrogen-free tubes with K_2_EDTA as an anticoagulant. Pericardial fluid (2–5 mL in K_2_EDTA and aprotinin) and a biopsy from the parietal sheet of the pericardium (2 x 2 cm) were obtained after median sternotomy and section of the pericardium at outset of the heart surgeries. The blood and pericardial fluid samples were centrifuged (3220 g for 10 min at 4°C) and the supernatants were stored at -80°C until analysis. The pericardial tissue biopsies were fixed in neutral buffered formalin for 48 hours at room temperature. Patients with haemolytic pericardial fluid samples were not further considered. Analyses were limited to the plasma, pericardial fluid and pericardial tissue biopsy from the same individual patients (N = 37). Routine plasma analyses for HbA1c, lipids, creatinine and high sensitivity C-reactive protein (hsCRP) were performed at the Odense University Hospital clinical biochemistry laboratory. Characteristics of the patients were disclosed to the researchers of the pericardial samples after they finalized their data collection. These characteristics are summarized in Table 1.

**Table 1:** Patient properties, plasma characteristics and prescribed medications.

**Property**	
N	37
CABG / valve / both (%)#	54 / 27 / 19
Age (years)	68 ± 9
Male (%)	84
Smoking Y / F / N (%)	14 / 49 / 38
BMI (kg/m^2^)	28.8 ± 4.9
Systolic BP (mmHg)	137 ± 21
Diastolic BP (mmHg)	74 ± 13
Ejection fraction (%)	55 (30–70)
Known hypertension (%)	70
Type 2 diabetes (%)	49
Dyslipidemia (%)	81
**Plasma concentrations**	
hsCRP (mg/L)	3.9 (1.8–8.4)
HbA1c (mmol/mol)	40 (35–48)
Total cholesterol (mmol/L)	3.6 (3.2–4.3)
LDL cholesterol (mmol/L)	1.9 (1.6–2.5)
HDL cholesterol (mmol/L)	1.1 (0.9–1.3)
Triglycerides (mmol/L)	1.43 ± 0.63
Creatinine (μmol/L)	90 (78–111)
**Medications**	
Aspirin (%)	65
Other anti-coagulant (%)	32
Statin (%)	76
ACEI /ARB (%)	41 / 16
Beta blocker (%)	60
Calcium antagonist (%)	32
Thiazide diuretic (%)	21
Loop diuretic (%)	27
Aldosterone antagonist (%)	14
Insulin (%)	14
Biguanide (%)	35
Sulfonylurea (%)	8

Categorical data are shown as % of the study group, normally distributed continuous variables as mean ± SD, non-normally distributed continuous variables as median value (interquartile range).

^#^, type of surgery. CABG, coronary artery bypass surgery; BMI, body mass index; BP, blood pressure; hsCRP, high sensitivity C-reactive protein; ACEI/ARB, inhibitor of angiotensin converting enzyme or antagonist of angiotensin AT1 receptors.

### Measurements of adipokine concentrations

The levels of APN and A-FABP in P and PF were measured by the respective sandwich ELISA kits (Antibody and Immunoassay Services (AIS), The University of Hong Kong, Hong Kong, China) as previously described [26, 27]. P and PF levels of leptin were determined with an ELISA kit from Biovendor Laboratories (Brno, Czech Republic) as previously described [27]. All samples were determined in duplicate. For one patient, plasma A-FABP was below the detection limit.

### Western blotting

For detection of different oligomeric complexes of APN, P and PF samples were incubated with nonreducing sample buffer (1% SDS, 5% glycerol, and 10 mM Tris-HCl (pH 6.8)) at room temperature for 10 min, separated by 4–20% gradient SDS-PAGE, transferred to polyvinylidene difluoride membranes, and immunoblotted with affinity-purified rabbit anti-human APN IgG (AIS; 0.2 μg/mL) as we described previously [28]. Based on the ELISA results, samples containing the same amount of total APN (20 ng) were analyzed by non-reducing SDS-PAGE and Western blotting for detection of the distribution of oligomers of APN. Quantification was performed by densitometric analysis using ImageJ software (National Institutes of Health, Bethesda, MA, USA). Increasing concentrations of recombinant human Flag-tagged APN protein purified from mammalian cells were used to generate the standard curves and to establish the specificity of the experimental approach in comparison to P and PF samples from 3 randomly selected patients (S1 Fig). Abundance of APN oligomers with molecular weight > 200 kDa was expressed as % of total APN.

### Immunohistochemistry

Presence and distribution of A-FABP and APN in pericardial tissue biopsies were determined by immunohistochemical (IHC) staining. Paraffin sections (5 μm) of formalin-fixed tissue were rehydrated and endogenous peroxidase activity was quenched with H_2_O_2_ in methanol. Microwave treatment was used for heat induced antigen retrieval using citrate (for APN) or Tris-ethylene glycol (TEG, for A-FABP). Sections were incubated overnight at 4°C with primary monoclonal antibody against APN (1:500, ab22554, ABCAM, Cambridge, UK) or polyclonal antibody against A-FABP (1:1200, 11030, AIS, Hong Kong, China) followed by incubation with goat anti-mouse or goat anti-rabbit secondary antibody, respectively (both 1:1000, P0447 and P0448 resp. Dako, Denmark) for 45 minutes. Colorimetric detection was completed with 3.3’-diaminobenzidine (DAB). Sections were counterstained with Mayer’s hematoxylin. Sections without primary antibody served as negative controls. Digital images were obtained with a microscope (Olympus BX51, Japan) and imaging software (cellSens Entry, Olympus Corporation, version 1.7). To estimate the size and adipokine content of adipocytes, histological sections were viewed at 10 x magnification and analyzed with ImageJ software (version 2.0.0-rc-23; NIH, Bethesda, MD, USA). Adipocyte cross-sectional area was determined as described by Chen and Farese [29]. The percentage of adipocytes staining for APN or A-FABP was evaluated in 3–50 square fields (60000 pixels) depending on the thickness of the paracardial adipose tissue.

### Statistics

Continuous variables that were normally distributed are shown as mean ± standard deviation (SD). Concentrations of biomarkers that were not normally distributed (Shapiro-Wilk normality test) are shown as median value and interquartile range (IQR). Paired comparisons between observations in P and PF samples of the same individuals were performed with Wilcoxon matched-pairs signed rank tests. Associations between the levels of adipokines in PF and P and between adipokine concentrations and clinical variables were evaluated with Pearson’s correlation tests after Log_10_ transformation of the data which resulted in normal distribution. Statistical analyses were performed with GraphPad Prism v6.05 (GraphPad Software Inc, San Diego, Ca, USA) and IBM SPSS (statistical package for the social sciences softwares; Chicago, Il, USA). Differences and associations were considered statistically significant at *p* < 0.05.

## Results

### Patient characteristics

Table 1 summarizes demographic, clinical and plasma characteristics of the study group. Patients were undergoing CABG (54%), cardiac valve replacement (27%) or both (19%) indicating that 73% were suffering from ischemic heart disease due to CAD. As expected, the majority were elderly overweight males (84%) with a high prevalence of systemic inflammation (high plasma hsCRP levels), dyslipidemia (81%), hypertension (70%) and type 2 diabetes (49%) but not overt heart failure or renal failure as judged from ejection fraction and plasma creatinine. Most had been prescribed a variable combination of anti-coagulant, cholesterol lowering, renin angiotensin system inhibitory, anti-hypertensive and anti-diabetic medications.

### Pericardial fluid levels of leptin, A-FABP and APN

**Fig 1:**
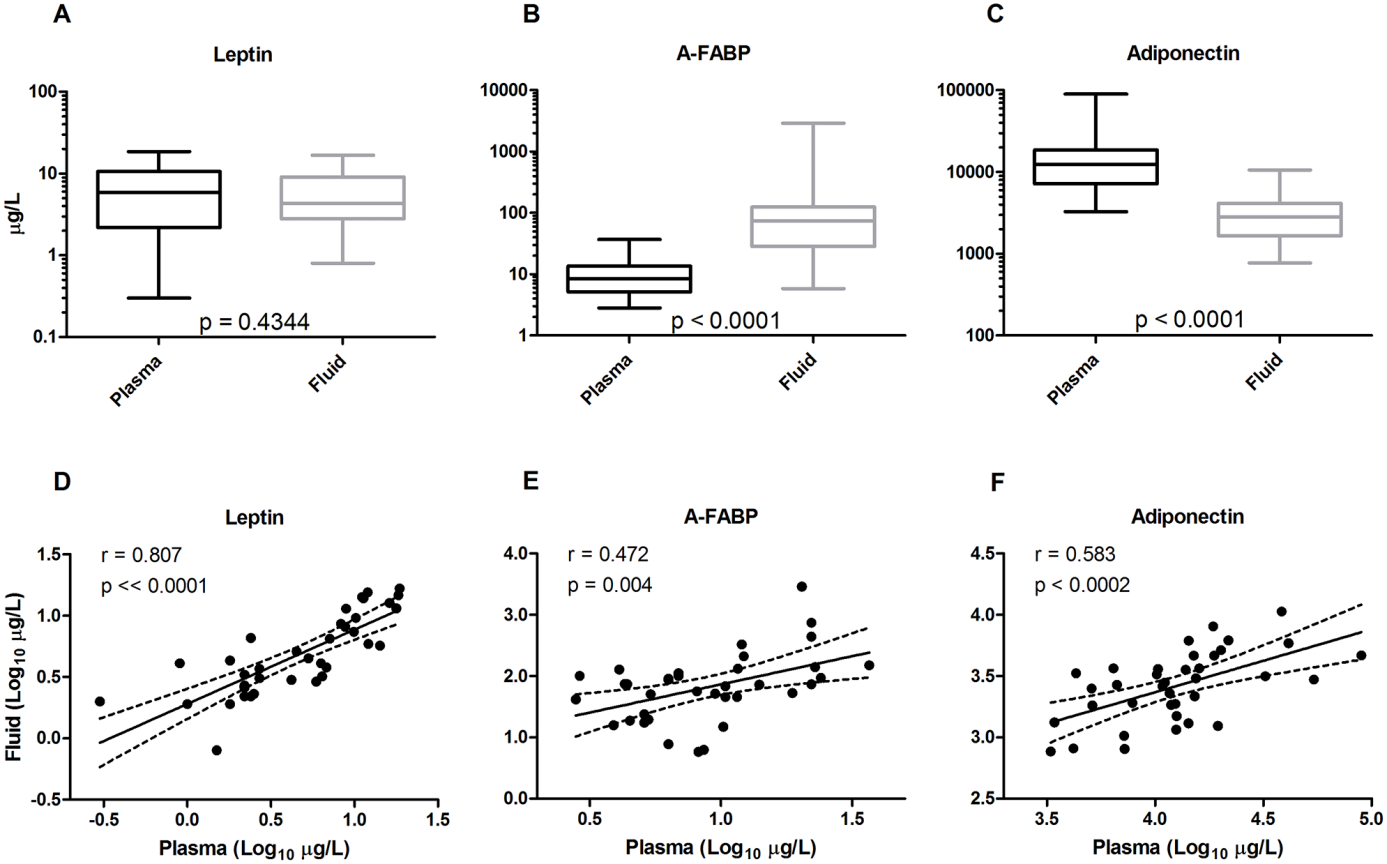
Venous plasma and pericardial fluid concentrations of leptin (left), adipocyte fatty acid-binding protein (A-FABP, middle) and adiponectin (right) in cardiac and vascular disease patients (n = 36–37). Top, box plots illustrating median values, interquartile range and range of concentrations compared by Wilcoxon matched-pairs signed rank test. Bottom, double logarithmic plots by Pearson’s correlation illustrating the relationship between pericardial fluid concentrations and circulating levels of the adipokines.

The concentrations of leptin in PF were comparable to those in the circulating P (4.3 (2.8–9.1) versus 5.9 (2.2–11) μg/L, *p* = 0.4344; Fig 1A). A-FABP, on the other hand, was 9 times more abundant in PF than P (73 (28–124) versus 8.4 (5.2–14) μg/L, *p* < 0.0001; Fig 1B) while total APN was 5 times less concentrated in PF than P (2.8 (1.7–4.2) versus 13 (7.2–19) mg/L, *p* < 0.0001; Fig 1C; Table 2). As a consequence, the leptin/APN ratio was significantly larger in PF than P (2.2 (1.2–3.1) versus 0.44 (0.15–0.82) μg/mg; 5 fold). For A-FABP the imbalance was even more marked. The A-FABP/APN ratio was on average more than 30 times larger in PF than in venous P (24 (9.4–70) versus 0.69 (0.35–1.5) μg/mg). Despite marked differences between the levels of especially A-FABP and APN in PF and P, the concentrations of each of leptin, A-FABP and APN in PF were positively correlated to those in the venous plasma (Fig 1D–1F).

**Table 2:** Plasma (P) and pericardial fluid (PF) levels of three adipokines and univariate analyses of their interrelationships and associations with patient properties.

	P Leptin	PF Leptin	P A-FABP	PF A-FABP	P APN	PF APN
**Concentration**						
μg/L	5.9	4.3	8.4	73^###^		
	(2.2–11)	(2.8–9.1)	(5.2–14)	(28–124)		
mg/L					13	2.8^###^
					(7.2–19)	(1.7–4.2)
**Patient property**						
CABG surgery	ns	ns	ns	ns	**	ns
Age	ns	ns	ns	ns	0.386*	ns
BMI	0.588***	0.498***	ns	ns	-0.454***	ns
Type 2 diabetes	*	*	ns	ns	ns	ns
**Plasma (P)**						
HbA1c	0.554***	0.574***	0.362*	ns	ns	ns
HDL cholesterol	ns	ns	ns	ns	0.483**	0.344*
Triglycerides	ns	ns	ns	ns	-0.454*	ns
Creatinine	ns	0.408*	0.577***	0.330*	ns	ns
Leptin	1	0.807***	0.565***	ns	ns	ns
A-FABP	0.565***	0.573***	1	0.472***	ns	ns
Adiponectin (APN)	ns	ns	ns	ns	1	0.583***
**Medications**						
Statin	ns	ns	*	ns	ns	ns
ACEI / ARB	**	**	***	*	ns	ns
Biguanide	ns	ns	ns	ns	ns	*

A-FABP, adipocyte fatty acid-binding protein; APN, total adiponectin; ns, not statistically significant; other abbreviations as in Table 1. Concentrations are shown as median (interquartile range);

^###^: *p* < 0.001 versus plasma by Wilcoxon matched-pairs signed rank test.

*, *p* < 0.05;

**, *p* < 0.01 and

***, *p* < 0.001 indicate statistically significant correlations to categorical or continuous variables. For continuous variables the Pearson’s correlation coefficient is shown as well.

**Fig 2:**
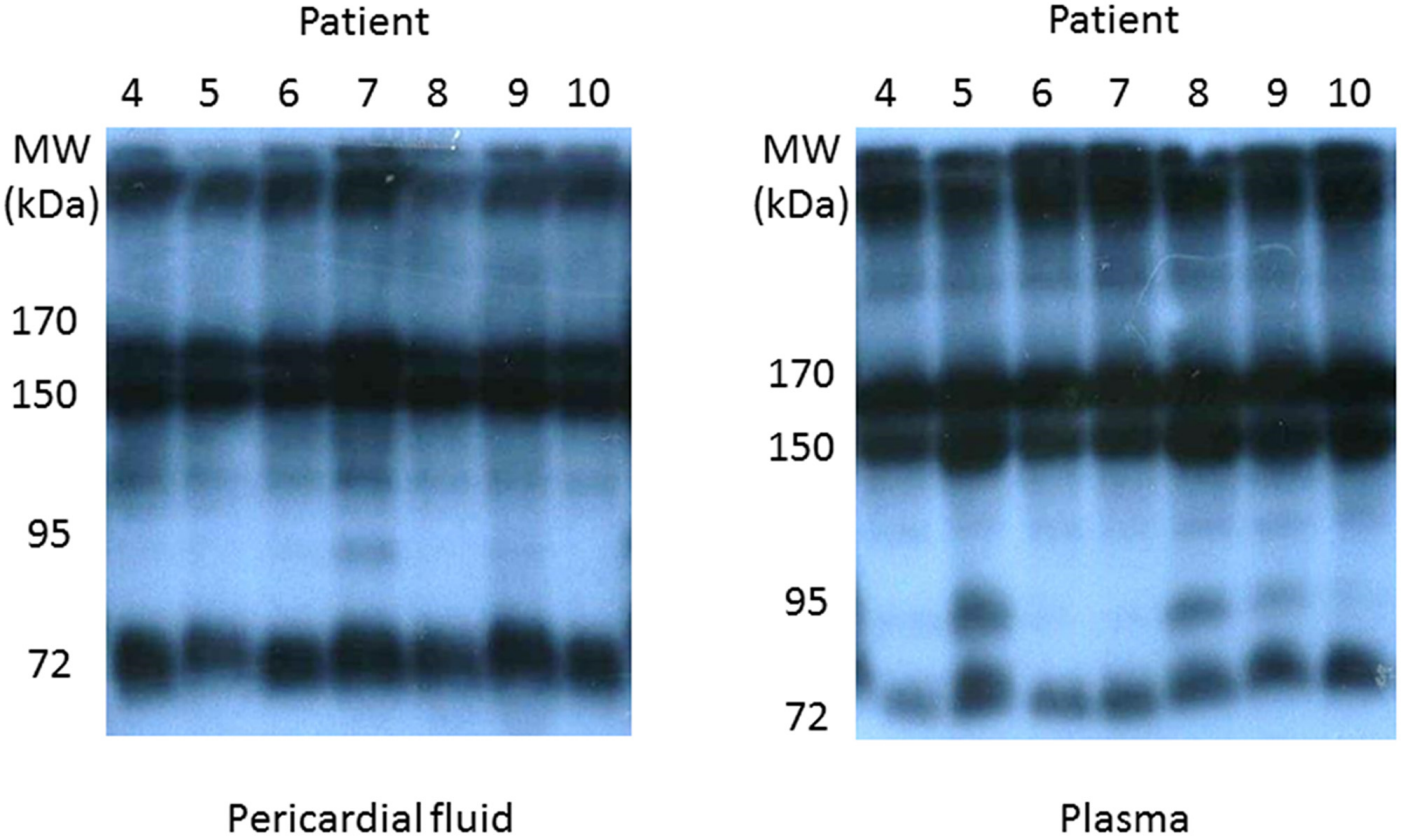
Representative Western blot images of APN multimers in pericardial fluid (left) and venous plasma (right) of cardiac and vascular disease patients. The Western blots illustrate the composition of oligomeric complexes of APN in pericardial fluid and plasma samples from the same 7 individual patients, indicated by numbers 4–10. The contribution of high molecular APN species (> 200 kDa) to the total concentration of APN was calculated as a percentage as described in the methods section.

Western blotting was used to determine the relative abundance of multimers of APN. Typical results for PF and P from the same individual patients are shown in Fig 2. The contribution of high molecular weight APN (molecular weight > 200 kDa) to total APN did not differ significantly between PF and venous P (29.1 ± 7.5 and 27.4 ± 6.8%, respectively).

### Paracardial adipose tissue properties

**Fig 3:**
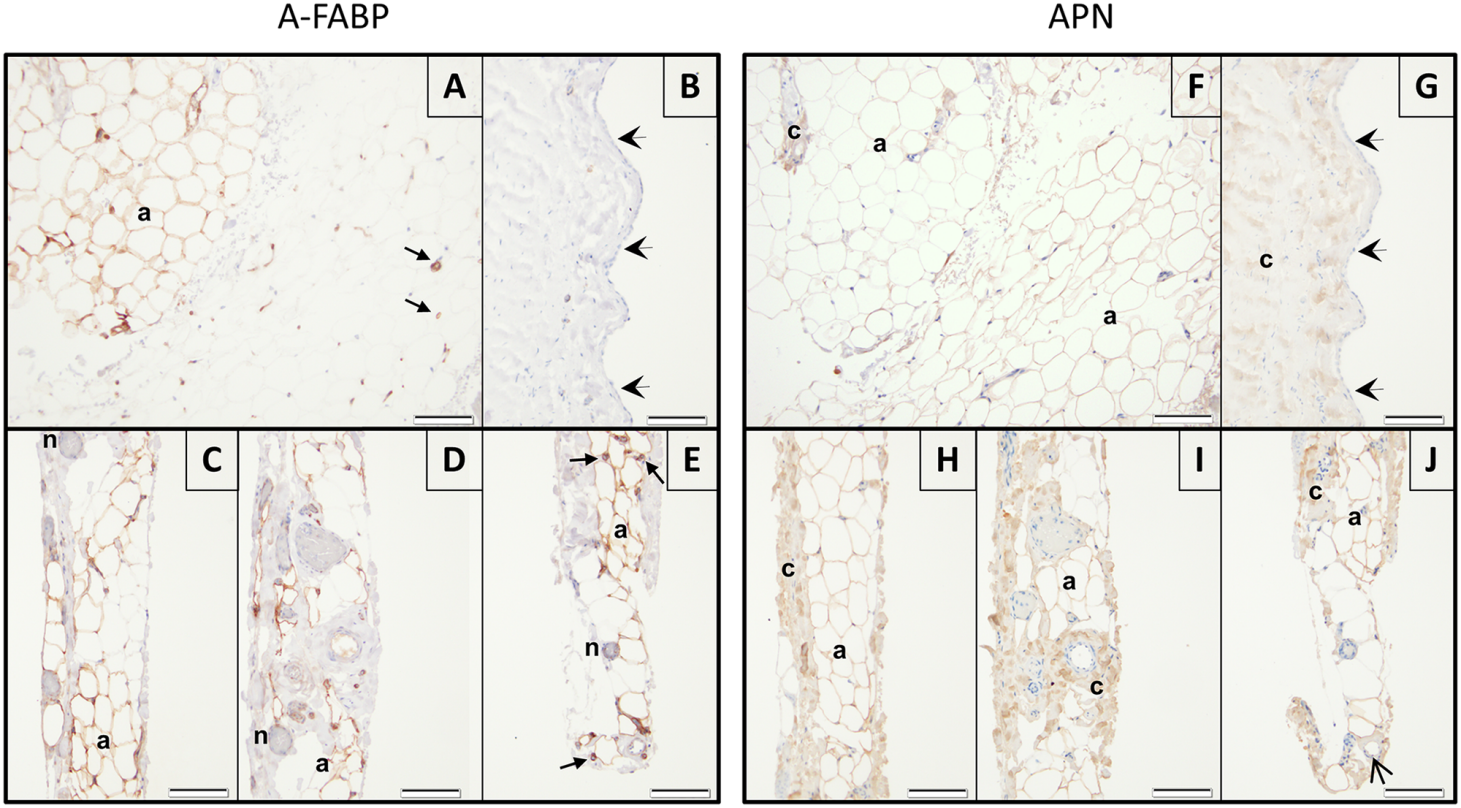
Representative images of immunohistochemical stainings of adipocyte fatty acid-binding protein (A-FABP; left) and adiponectin (APN; right) in the parietal pericardium. Top, adjacent sections of the adipose tissue part (A and F) and of the luminal (mesothelial) side (B and G) from the same biopsy. Bottom, adjacent sections of the biopsy from another patient focusing on the sub-mesothelial vasculature and its peri-vascular adipose tissue (C-E and H-J). Positive immunoreactivity in the different structures are indicated as follows: a = adipocytes, n = nerves, c = collagen rich regions, open arrow head = endothelium, small arrows = arterioles and capillaries. The thick arrow heads indicate mesothelial cells. Scale bars, 100 μm.

Fig 3 illustrates structural features and distribution of A-FABP and APN in the parietal sheet of the pericardium. Mesothelial cells that line the pericardial cavity lay on a thick collagen band. Abluminally from this structure, a vascular network [30] and paracardial adipose tissue are located. The thickness of this adipose tissue differed markedly between patients. The median cross sectional area of paracardial adipocytes was 12 (8.0–17) 102 μm2. With IHC staining, APN was found in parts of the adipose tissue, vascular endothelium (some patients only), and in collagen-rich regions. A-FABP was located in some of the adipocytes, nerves, arterioles and capillaries. Mesothelium and arterial smooth muscle cells did not stain for A-FABP or APN. Presence of detectable A-FABP and APN in adipocytes displayed marked regional heterogeneity (Fig 2). The percentage of paracardial adipocytes that was immunoreactive for A-FABP or APN varied considerably between patients (43 (0.0–94) and 63 (14–100) %), respectively).

### Interrelationships and associations

In PF, there were no statistically significant relationships between the levels of the adipokines investigated while in P the levels of leptin and A-FABP were positively correlated (Table 2). As expected [1, 31], the circulating level of leptin increased with increasing BMI while the circulating concentration of APN was inversely correlated to BMI (Table 2). Also in PF, leptin levels were positively correlated to BMI but the concentrations of APN in PF displayed no significant relation to BMI.

Table 2 further summarizes associations between the concentrations of adipokines and patient characteristics listed in Table 1. The concentrations of leptin in both P and PF were each related to type 2 diabetes, BMI, circulating levels of glycated hemoglobin and to treatment with an inhibitor of the renin-angiotensin system. For A-FABP in P and PF there were fewer similarities. This discrepancy between the adipokine concentrations in the two compartments was even more marked for APN. APN in P, but not PF, was inversely related to CAD, BMI, and plasma triglycerides and positively correlated to the age of the patients (Table 2).

The size of the paracardial adipocytes was significantly larger in type 2 diabetic patients (13.7 (11.1–18.0) versus 10.2 (6.6–13.1) x 10^2^ μm^2^, *p* = 0.038) and in CAD patients (13.6 (10.7–18.0) versus (6.7 (5.9–9.9) x 10^2^ μm^2^, *p* = 0.0004) but was not correlated to BMI (*p* = 0.642, *r* = 0.0839), not related to the PF level of A-FABP (*p* = 0.735, *r* = 0.0611) and inversely related to the PF level of APN (*p* = 0.0364, *r* = -0.366). Paracardial adipocyte immunoreactivity for A-FABP or APN were significantly correlated (*p* = 0.0469, *r* = 0.3720) but not significantly related to adipocyte size (*p* = 0.352, *r* = -0.179 and *p* = 0.223, *r* = -0.218 respectively) or to the PF concentration of A-FABP or APN (*p* = 0.507, *r* = 0.124 and *p* = 0.460, *r* = 0.133, respectively).

## Discussion

In pericardial fluid of cardiothoracic surgery patients, the concentration of leptin was comparable but the level of A-FABP markedly higher and that of APN considerably lower than in the circulating plasma. Leptin levels in pericardial fluid and plasma were correlated to most of the same patient characteristics while APN in pericardial fluid was unlike in the plasma not correlated to age, body mass index, type 2 diabetes, plasma triglycerides and CAD. The concentration of APN but not A-FABP in pericardial fluid was related to the size of paracardial adipocytes, whereas both adipokine concentrations in pericardial fluid were not related to the immunoreactivity of the adipocytes in the paracardial pericardium. These findings indicate that the pericardial cavity of cardiovascular disease patients is a pro-inflammatory atherogenic adipokine microenvironment determined by components of the heart rather than by the circulating hormones or paracardial adipose tissue.

Increased risk for metabolic and cardiovascular diseases associated with increased adiposity [1, 2] stimulated interests in signaling molecules secreted by adipose tissues [4–7]. These adipokines can reach the cardiovascular system as hormones via the circulation and as paracrine mediators produced by comparatively small adipose tissues that surround the heart and blood vessels. Hyperleptinemia and hypoadiponectinemia in obesity are strongly associated with type 2 diabetes but only moderately with CAD [32, 33]. Circulating levels of A-FABP increase with the number of stenotic coronary arteries [34]. On the other hand, the volume of EAT correlates positively with CAD in not only obese but also non-obese patients [8, 9, 15, 17]. Moreover, several classical vasoactive mediators and angiogenic growth factors are more abundant in the pericardial fluid than in the circulation of ischemic heart disease and heart failure patients [21, 23–25]. For these reasons we performed a small paired cross sectional comparison of concentrations of leptin, A-FABP and APN in pericardial fluid and venous plasma. Pericardial fluid was obtained at outset of coronary artery bypass or cardiac valve replacement surgery where the parietal pericardium must be opened to gain access to the heart. The characteristics of the patients that we investigated are comparable to those previously studied with respect to EAT volume and histology [10, 11, 15, 16, 18, 20] and coronary arteriolar endothelial dysfunction [35, 36]. Pericardial fluid was compared to a venous blood sample from the day before surgery. This is justified because earlier studies found no changes in the circulating levels of leptin and APN and an at most 50% increase of plasma A-FABP during the early steps of cardiothoracic surgery [37, 38].

Experimental animal evidence (reviewed by Beltowski [5]) links elevations in circulating levels of the 16 kDa protein leptin via loss of leptin function or leptin resistance to atherogenesis and CAD but findings in human patients are less convincing [32]. In line with earlier reports [5, 31, 39, 40] we observed that in human patients the circulating levels of leptin are positively correlated to BMI, type 2 diabetes and consequences thereof such as elevated HbA1c, but not CAD, age or plasma cholesterol and triglycerides. We found that leptin in pericardial fluid, which to the best of our knowledge was not reported before, is comparable to that in the venous plasma in terms of not only concentration but also relation to most of the same patient characteristics. In sharp contrast to leptin, marked quantitative and qualitative differences were observed for A-FABP and APN. Experimental animal evidence (reviewed by Xu and Vanhoutte [6]) links circulating levels of the 15 kDa protein A-FABP that can be secreted by adipocytes and macrophages, to cardiovascular diseases and findings in patients link it to the severity of CAD [34] and heart failure [27]. In our paired comparison, A-FABP was nine times more abundant in pericardial fluid than in circulating plasma and while plasma concentrations were significantly related to plasma HbA1c and leptin levels and to treatment with statins, pericardial fluid concentrations were not. Experimental evidence (reviewed by Hui et al. [4], Xu and Vanhoutte [6] and Mattu and Randeva [7]) links reductions of the circulating levels of the 90 (trimeric), 180 (hexameric) and >250 kDa protein APN to endothelial dysfunction and atherogenesis. Again the data available for patients are less convincing [33]. In our study, we confirmed the inverse relationships in CVD patients between circulating total APN on the one hand and CAD, BMI and plasma triglycerides on the other hand and the positive relation between circulating total APN with age and plasma HDL cholesterol described in earlier reports [4, 6, 31, 41]. Levels of APN in patient pericardial fluid, which were recently determined but not correlated to circulating plasma levels [10], were in our hands 4.5 times lower than in the circulation and not significantly related to any of the factors that seem to govern plasma APN levels with the exception of a weak association with plasma HDL cholesterol. Combined our findings indicate that the adipokine composition in pericardial fluid of cardiac and vascular diseases patients differs quantitatively and qualitatively from that in the circulation. For instance, the leptin/APN and the A-FABP/APN ratio’s that are advanced as powerful diagnostic markers [41–43] are 5 and more than 30 times larger in the local pericardial fluid than in the circulation, respectively. Although adipokine levels differed markedly between both compartments, the circulating and pericardial concentrations of leptin, A-FABP and APN were strongly correlated. Thus plasma concentrations may be considered in future longitudinal studies estimating local pericardial changes of adipokines during progression and treatment of cardiac and coronary artery diseases. These will at best be qualitative in nature and underestimate imbalances between adipokine species.

The pericardial cavity is considered for local treatment of ischemic heart disease and myocardial infarcts by drugs, angiogenic growth factors and stem cells [44, 45]. Control of pericardial fluid composition is however poorly understood. Some authors consider it a protein poor ultrafiltrate of the plasma [46, 47] while others emphasize similarities to the interstitium of the heart [21, 22, 47]. Our observations of comparable contributions of low and high molecular weight APN isoforms in plasma and pericardial fluid and of different pericardial to plasma ratios for leptin and A-FABP along with earlier findings of elevated levels of lactate dehydrogenase (140 kDa) in pericardial fluid [46], are not compatible with the former view. We considered contributions of the paracardial adipose tissue lining the outer surface of the parietal pericardium. Paracardial adipocyte size was enlarged in diabetic and CAD patients as previously reported for epicardial adipocytes [48]. Mean paracardial adipocyte immunoreactivities for A-FABP and APN were interrelated but not related to adipocyte size in contrast to the negative associations between the size of subcutaneous and epicardial adipocytes and APN gene expression previously observed by Bambace et al [48]. We could not observe a significant relationship between mean paracardial adipocyte immunoreactivity and pericardial fluid levels of A-FABP and APN. This could indicate that there is no major contribution of paracardial adipose tissue to pericardial fluid levels of adipokines. For the two adipokines that we stained in parietal pericardium we observed marked regional heterogeneities. Thus in addition to differences between adipose tissues at different anatomical locations [1, 16–20, 49], adipokine biology seems to differ locally within a given adipose tissue. It will be of interest to verify in future investigations whether these regional heterogeneities are correlated to distances from blood vessels, capillaries or macrophages, in order to discriminate between differences from perivascular adipose tissue and influences of local oxygen tension and inflammation.

### Study Limitations

In this cross sectional investigation of randomly selected cardiothoracic surgery patients a fair number of strong associations could be observed. The causal nature of these could not be addressed. The study was too small to appreciate influences of the more rare aspects in the study population such as female sex and specific medications, or to perform multivariate analysis. No pericardial fluid could be sampled from individuals without CVD or cardiovascular drug treatments. This may be partly remedied by studies in a sufficiently large relevant experimental animal model [50] and sampling of pericardial fluid from patients undergoing thoracic surgery for non-CVD [51]. Confounding influences of drugs such as statins and PPARγ agonists that have been observed to influence circulating levels and local expressions of adipokines [3, 4, 14] might be more difficult to deal with. At this stage we addressed only three proteins from a broad spectrum of candidate adipokines [1, 7, 17, 31] and future studies may want to address a broader panel of adipocyte derived signaling molecules. Our suggestions about the lack of relation between paracardial adipose tissue and pericardial fluid adipokine content are weakened by the absence of information on paracardial tissue volume which is not part of the diagnostics of our elective cardiothoracic surgery patients. More importantly, we lack information on epicardial adipose tissue volume [9, 11–13, 15, 16], immunohistochemistry and adipokine secretion [14, 16, 17, 19] to propose this adipose tissue compartment as a determinant of pericardial fluid adipokine composition. Finally, whether the pericardial fluid adipokine profile i) represents spillover from the cardiac interstitium with potential for biomarker research of epicardial adipose tissue effects on CAD and ii) might in itself contribute to coronary atherogenesis, coronary microvascular dysfunction, arrhythmogenesis and heart failure, will require a broad variety of dedicated investigations.

## Supporting Information

S1 Fig Standard curve and specificity of adiponectin protein detection by Western blotting. (DOCX)
