# Supplementary material for: Adipokine Imbalance in the Pericardial Cavity of Cardiac and Vascular Disease Patients
Source: PLoS One. 2016 May 3;11(5):e0154693. doi: 10.1371/journal.pone.0154693 (PMC4854456; doi:10.1371/journal.pone.0154693)
Supplement: S1 Fig — (DOCX) [file pone.0154693.s001.docx]

**S1. Standard curve and specificity of adiponectin protein detection by Western blotting.**

**
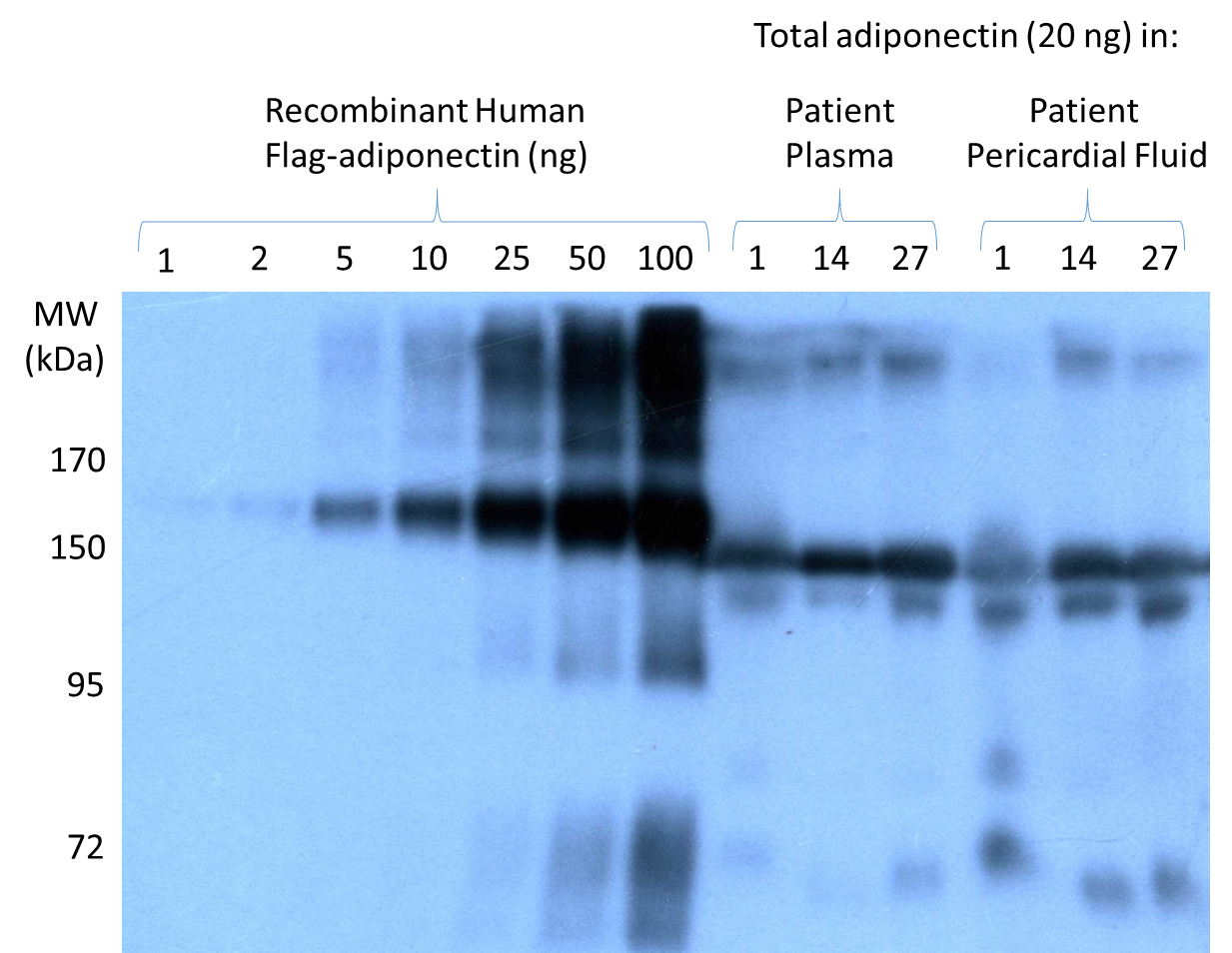
**

**Detection of multimers of recombinant human adiponectin and of adiponectin in plasma and pericardial fluid.** Increasing amounts of purified Flag-tagged recombinant human adiponectin (left; 1 - 100 ng) were used to generate the standard curve and to establish specificity of the method. On the right hand side, plasma and pericardial fluid samples from 3 randomly selected patients (indicated by numbers 1, 14 and 27) each containing 20 ng of total adiponectin protein (determined by ELISA), are depicted.
